# Buspirone Counteracts MK-801-Induced Schizophrenia-Like Phenotypes through Dopamine D_3_ Receptor Blockade

**DOI:** 10.3389/fphar.2017.00710

**Published:** 2017-10-04

**Authors:** Sebastiano Alfio Torrisi, Salvatore Salomone, Federica Geraci, Filippo Caraci, Claudio Bucolo, Filippo Drago, Gian Marco Leggio

**Affiliations:** ^1^Department of Biomedical and Biotechnological Sciences, School of Medicine, University of Catania, Catania, Italy; ^2^Department of Drug Sciences, University of Catania, Catania, Italy; ^3^Oasi Institute for Research on Mental Retardation and Brain Aging (IRCCS), Troina, Italy

**Keywords:** buspirone, dopamine D_3_ receptor, MK-801, schizophrenia, antipsychotics, prepulse inhibition, temporal order recognition

## Abstract

**Background:** Several efforts have been made to develop effective antipsychotic drugs. Currently, available antipsychotics are effective on positive symptoms, less on negative symptoms, but not on cognitive impairment, a clinically relevant dimension of schizophrenia. Drug repurposing offers great advantages over the long-lasting, risky and expensive, *de novo* drug discovery strategy. To our knowledge, the possible antipsychotic properties of buspirone, an azapirone anxiolytic drug marketed in 1986 as serotonin 5-HT_1A_ receptor (5-HT_1A_R) partial agonist, have not been extensively investigated despite its intriguing pharmacodynamic profile, which includes dopamine D_3_ (D_3_R) and D_4_ receptor (D_4_R) antagonist activity. Multiple lines of evidence point to D_3_R as a valid therapeutic target for the treatment of several neuropsychiatric disorders including schizophrenia. In the present study, we tested the hypothesis that buspirone, behaving as dopamine D_3_R antagonist, may have antipsychotic-like activity.

**Materials and Methods:** Effects of acute administration of buspirone was assessed on a wide-range of schizophrenia-relevant abnormalities induced by a single administration of the non-competitive NMDAR antagonist MK-801, in both wild-type mice (WT) and D_3_R-null mutant mice (D_3_R^-/-^).

**Results:** Buspirone (3 mg⋅kg^-1^, i.p.) was devoid of cataleptogenic activity in itself, but resulted effective in counteracting disruption of prepulse inhibition (PPI), hyperlocomotion and deficit of temporal order recognition memory (TOR) induced by MK-801 (0.1 mg⋅kg^-1^, i.p.) in WT mice. Conversely, in D_3_R^-/-^ mice, buspirone was ineffective in preventing MK-801-induced TOR deficit and it was only partially effective in blocking MK-801-stimulated hyperlocomotion.

**Conclusion:** Taken together, these results indicate, for the first time, that buspirone, might be a potential therapeutic medication for the treatment of schizophrenia. In particular, buspirone, through its D_3_R antagonist activity, may be a useful tool for improving the treatment of cognitive deficits in schizophrenia that still represents an unmet need of this disease.

## Introduction

Schizophrenia is a chronic and devastating multifactorial mental illness affecting approximately 0.7–1% of population worldwide (Landek-Salgado et al., 2016). The development of second-generation antipsychotics has yielded some advances in terms of efficacy, but only modest improvement in addressing the negative symptoms of schizophrenia. To date, no antipsychotics display robust effects on cognitive deficits or impaired social processing that are the most clinically relevant dimensions of the disease (Owen et al., 2016). Drug repositioning refers to the process of finding new uses for already approved and commercialized medications and it is thought to offer great advantages over the long-lasting, risky and expensive *de novo* drug discovery strategy. This is because the pharmacological and toxicological profiles of approved medications are well-characterized (Ashburn and Thor, 2004). It has been suggested that repositioned drugs may represent effective alternative compounds for the treatment of neuropsychiatric disorders for which the classical drug discovery process is hampered by the poor knowledge of the pathophysiological mechanisms (Lee and Kim, 2016). In this context, the azapirone anxiolytic drug buspirone (Buspar^®^), has been proposed for the treatment of substance use disorder (SUD; Leggio et al., 2016). Regarding schizophrenia, earlier clinical trials suggested that buspirone added to both typical and atypical antipsychotics ameliorates negative symptoms (Ghaleiha et al., 2010; Sheikhmoonesi et al., 2015), while other preclinical and clinical data showed buspirone as scarcely effective in improving cognitive dysfunction (Piskulić et al., 2009; Horiguchi and Meltzer, 2012; Maeda et al., 2014).

At a pharmacological level, buspirone, besides its claimed 5-HT_1A_R partial agonist activity, is endowed with D_3_R/D_4_R antagonist activity and binds to dopamine D_2_ receptor (D_2_R) with an affinity 5-fold lower than for D_3_R (Bergman et al., 2013). Available evidence indicates that D_3_R can be considered as a new validated pharmacological target for the treatment of several neuropsychiatric disorders, including SUD, Parkinson’s disease, depression and schizophrenia (Leggio et al., 2016; Maramai et al., 2016; Sokoloff and Le Foll, 2017). Published studies indicate that D_3_R play a key role in the pathophysiology of schizophrenia (Nunokawa et al., 2010; Gross et al., 2013). Moreover, D_3_R expression is increased in schizophrenics (Gurevich et al., 1997; Cui et al., 2015). The restricted localization of D_3_Rs in the limbic system, particularly in the nucleus accumbens (NAc), has attracted great interest especially for the development of safe and effective medications devoid of the classical side effects (extrapyramidal side effects and prolactin elevation) caused by D_2_R blockade (Gross and Drescher, 2012). In fact, antipsychotics targeting D_3_R, such as blonanserin and cariprazine, have been demonstrated effective in treating positive and negative symptoms with a good safety profile (Hori et al., 2014; Leggio et al., 2016; Earley et al., 2017). Beside the high expression in NAc, D_3_Rs are expressed specifically in the layer 5 pyramidal neurons of medial prefrontal cortex (mPFC, Lidow et al., 1998), where they control in a peculiar manner neuronal excitability (Clarkson et al., 2017). D_3_Rs play a fundamental role in physiological mechanisms underlying mPFC-dependent cognitive functions as well as in crucial pathophysiological processes subserving mPFC-dependent cognitive dysfunctions (Nakajima et al., 2013). In particular, it seems that selective antagonism on D_3_R improves cognitive functions while selective agonism exerts opposite, detrimental effects (Watson et al., 2012). Recently, it has been proposed that molecules joining 5HT_1A_R partial agonism and 5-HT_2A_ antagonism to D_3_R antagonism may exhibit stronger antipsychotic effects (Brindisi et al., 2014). As aforementioned, the pharmacological profile of buspirone largely matches that of these potential antipsychotics. However, as far as we know, the antipsychotic properties of buspirone have not yet been extensively elucidated.

In the present study, we tested the hypothesis that buspirone, behaving as dopamine D_3_R antagonist, may exert antipsychotic-like properties in a preclinical model of schizophrenia, based on NMDAR hypofunction. This pharmacological model, as compared with dopamine-based models, appears to more efficiently recapitulate several symptoms of schizophrenia, particularly those related to cognitive dysfunction (Kantrowitz and Javitt, 2010). The effect of acute administration of buspirone was evaluated on hyperlocomotion, prepulse inhibition (PPI) disruption and temporal order recognition (TOR) memory impairment, elicited by acute administration of the non-competitive NMDAR antagonist MK-801 in WT mice. In order to assess the involvement of D_3_R on the effect of buspirone, the same behavioral paradigms, with or without buspirone, were applied to D_3_R^-/-^ mice.

## Materials and Methods

### Animals and Housing

In these experiments, D_3_R^-/-^ mice and their WT littermates (males, 8–12 weeks old), bred by a heterozygous (D_3_R^+/-^ × D_3_R^+/-^) mating strategy, were tested. Animals were group-housed (2–5 mice per cage), with free access to chow and water, in an air-conditioned room, with a 12-h light–dark cycle. D_3_R mutant mice were 10th–12th generation of congenic C57BL/6J mice, generated by a back-crossing strategy (Accili et al., 1996). Genotypes were identified by PCR analysis of tail DNA as previously described (Leggio et al., 2011, 2015). The experimenters handled animals on alternate days during the week preceding the behavioral tests. Animals were acclimatized to the testing room at least 1 h before the beginning of the tests. Experiments were performed during the dark phase. All experimental procedures were carried out according to the Directive 2010/63/EU and were approved by the Institutional Animal Care and Use Committee of the Catania University.

### Drugs

(+)MK-801 hydrogen maleate and buspirone hydrochloride (Sigma–Aldrich, St. Louis, MO, United States) were dissolved in saline. Clozapine and haloperidol (Sigma–Aldrich) were dissolved in few drops of 1 N HCl and further diluted with saline; the pH was adjusted to 7 with NaHCO_3_. All drug solutions were prepared daily and intraperitoneally (i.p.) administered by using an injection volume of 10 ml/kg.

### Behavioral Testing

#### Temporal Order Recognition (TOR) Test

**FIGURE 1:**
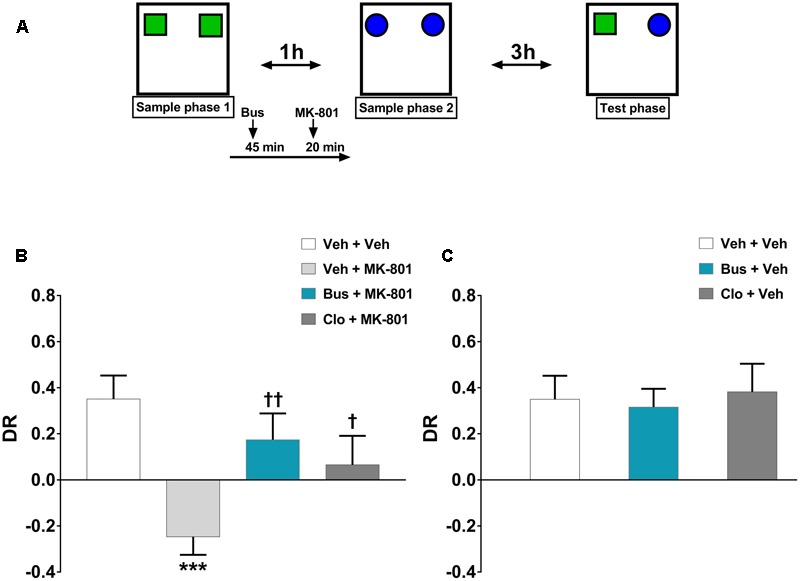
Buspirone counteracted MK-801-induced TOR memory impairment in WT mice. **(A)** Cartoon illustrating the TOR test and the schedule of treatment. Buspirone (Bus, 3 mg⋅kg^-1^, i.p.) or vehicle (Veh), clozapine (Clo, 1 mg⋅kg^-1^, i.p.) or Veh and MK-801 (0.1 mg⋅kg^-1^, i.p.) or Veh were injected 45, 30, and 20 min respectively, before the sample phase 2. **(B,C)** Discrimination ratio (DR) displayed by Veh + Veh (*n* = 10), Veh + MK-801 (*n* = 10), Bus + MK-801 (*n* = 9), Clo + MK-801 (*n* = 9), Bus + Veh (*n* = 8), Clo + Veh (*n* = 5) WT mice during the test phase. Data are shown as mean ± SEM. DR [(less recently experienced object exploration time - more recently experienced object exploration time)/total exploration time]. ^∗∗∗^*p* < 0.001 vs. Veh + Veh WT mice; ^††^*p* < 0.01 and ^†^*p* < 0.05 vs. Veh + MK-801 WT mice (One-way ANOVA and Newman–Keuls *post hoc* test).

The TOR test was carried out as previously described (Barker and Warburton, 2011; Managò et al., 2016) with minor modifications. Animals explored in an evenly illuminated (9 ± 1 lux) square open field (40 × 40 × 40 cm, Ugo Basile, Gemonio, Italy) in which the floor was covered with sawdust. The behavior of animals was recorded using a video camera (Sony Videocam PJ330E) and then scored by an independent observer. The objects presented were made of plastic Duplo blocks (Lego^®^), different in shape, color, and size (9 × 8 × 7 cm to 12 × 11 × 10 cm) and too heavy to be moved by the mice. After 1 week of handling, a 4-day pretesting procedure was carried out. Mice were habituated to the empty arena for 10 min on the day 1 and 2. Afterward, on the day 3 and 4, mice were i.p. injected with saline 20 min before being placed in the arena containing two objects (different from those ones eventually used during the test) for 10 min. This pretesting procedure was performed in order to minimize stress-related behavior induced by injections as well as to prevent neophobia during the test. The objects were located in two corners of the arena, 10 cm far from the sidewalls. The test consisted of two sample phases and one test trial (**Figure 1A**). During the sample phases, animals were allowed to explore two copies of an identical object for a total of 5 min. Different objects were used for each sample phase, with a delay between the sample phases of 1 h. The test trial was performed 3 h after the sample phase 2. During the test trial (5 min duration), animals were exposed to a third copy of the objects from sample phase 1 and a third copy of the objects from sample phase 2. Objects were cleaned with a 10% ethanol solution in between each test in order to avoid olfactory cues. Any feces were removed and the sawdust was shaken in order to equally redistribute any odor cues. If the temporal order memory is intact, animals should spend more time exploring the object from sample 1, the less recently experienced object, compared with the object from sample 2, the more recently experienced object. The objects utilized in each sample phase as well as the positions of the objects during the test were counterbalanced between the animals. Exploratory behavior was defined as the animal directing its nose toward the object at a distance of <2 cm. Looking around while sitting, climbing or rearing against the objects were not considered as exploration. Animals that failed to complete a minimum of 2 seconds (sec) of exploration in each phase of the task were excluded from the analysis. Discrimination between the objects was calculated using a discrimination ratio (DR) that takes into account individual differences in the total amount of exploration. In particular, data are depicted as DR, calculated as [(less recently experienced object exploration time – more recently experienced object exploration time)/total exploration time]. The higher is the DR, the better is TOR memory.

#### Acoustic Startle Response and Prepulse Inhibition (PPI) Test

Acoustic startle response and PPI were measured using four PPI sets from SR-Lab Systems (San Diego Instruments, San Diego, CA, United States). The experimental procedure was adapted from Papaleo et al. (2012). Animals were exposed to a short “matching” startle session before the PPI testing. They were placed in the startle chambers for a 5-min acclimation period with a 65 dB(A) background noise, and then exposed to a total of 17 acoustic startle stimulus (pulse) trials [40 ms — 120 dB (A) noise bursts] that were interspersed with 3 acoustic prepulse plus acoustic pulse trials in which the pulse was preceded 100 ms (onset-to-onset) by a 20 ms noise burst, 10 dB above background. Animals were assigned to each drug dose group based on average PPI% from the matching session to ensure similar baseline PPI levels between experimental groups. The PPI test session, with or without drug treatment, was carried out 5–7 days after the matching session. The animals were placed in the startle chambers for a 5-min acclimation period with a 65 dB(A) background noise. Animals were then exposed to a series of trial types, which were presented in pseudorandom order. The inter trial interval (ITI) was 5–60 s. One trial type measured the response to no stimulus (baseline movement), and another one measured the startle stimulus alone (acoustic amplitude), which was a 40 ms 120 dB sound burst. Other five trial types were acoustic prepulse plus acoustic startle stimulus trials. Prepulse tones were 20 ms at 70, 75, 80, 85, and 90 presented 100 ms before the startle stimulus. PPI was calculated by using the following formula: 100 ×{[pulse-only units - (prepulse + pulse units)]/(pulse-only units)}.

#### Open Field (OF) Test

Animals were tested in the same square open field mentioned above (divided into 16 quadrants by lines on the floor) over a 30 min-period. Locomotor activity was assessed during the first exposure to the empty open field arena. The apparatus was cleaned with a 10% ethanol solution in between each test to prevent olfactory cues. Locomotor activity was quantified by counting the numbers of lines crossed (crossings) with all four paws (Accili et al., 1996). The behavior of animals was recorded by using a video camera and eventually analyzed by one observer blinded to genotype/treatment.

#### Catalepsy Test

The catalepsy test was carried out as previously reported with minor changes (Fink-Jensen et al., 2011). The apparatus was made of 2 wooden supports linked by a steel bar (length: 7.5 cm; diameter 0.9 cm); The system was stabilized by another wooden support opposite to the steel bar. The catalepsy was evaluated by placing the animals with the forepaws on the horizontal steel bar positioned 4.5 cm above the floor. Animals were tested at different time points: 30, 60, 90, and 120 min after the pharmacological treatment. The latency (cut off time) was 600 s. The end point of the test was considered when both forepaws were removed from the bar or when the animal moved its head in exploratory manner. Each trial was repeated for three times and the highest time value was taken.

### Experimental Design

The behavioral effects of a single injection of buspirone were evaluated on MK-801-induced schizophrenia-like phenotypes both in WT and in D_3_R^-/-^ mice. These effects were compared to those of clozapine, the most effective commercially available antipsychotic (Owen et al., 2016), injected at a dose of 1 mg⋅kg^-1^. This dose has been revealed to be effective in ameliorating cognitive dysfunction (Mutlu et al., 2011; Park et al., 2014). The dose of buspirone (3 mg⋅kg^-1^) was selected based on our previous experience (Leggio et al., 2014) as well as according to a work by Di Ciano et al. (2017). To avoid effects of test-related anxiety, animals were divided into independent cohorts and subjected to the most stressful tests as the last. Animals were tested as follows: WT, cohort 1, open field test, catalepsy test; WT, cohort 2, TOR test, PPI test; D_3_R^-/-^, cohort 2, open field, TOR test. A washout period of at least 7 days was given between each experimental procedure.

#### Experiment 1 - Effect of Buspirone on MK-801-Induced TOR Memory Deficit in WT Mice

Administration of NMDAR antagonists before the sample phase 2 impairs TOR memory affecting both reconsolidation and consolidation mechanism (Warburton et al., 2013). Therefore, buspirone, clozapine and MK-801 were administered 45, 30, and 20 min, respectively, before the sample phase 2. The chosen dose of MK-801 (0.1 mg⋅kg^-1^) is able to produce cognitive impairment without inducing locomotor disturbance (stereotypies, ataxia; Blot et al., 2015).

#### Experiment 2 - Effect of Buspirone on MK-801-Stimulated Hyperlocomotion and Assessment of Catalepsy in WT Mice

Mice received injections of buspirone, clozapine and MK-801 with the same timing of treatment used for the TOR test and then placed into the empty open field. The dose of 0.1 mg⋅kg^-1^ MK-801 is effective in stimulating hyperlocomotion (Zhang et al., 2007). For the catalepsy test, animals were injected with buspirone, clozapine and haloperidol (1 mg⋅kg^-1^) and then tested at different time points (30, 60, 90, and 120 min). The haloperidol-induced catalepsy at the dose of 1 mg⋅kg^-1^ is a widely used model for the evaluation of extrapyramidal side effects induced by drugs (Pogorelov et al., 2017).

#### Experiment 3 - Effect of Buspirone on MK-801-Induced PPI Disruption in WT Mice

Mice were given injections of buspirone, clozapine and MK-801, 45, 30, and 20 min (including the 5-min acclimation period), respectively, before to be placed in the startle chambers for the PPI test. We chose the dose of 0.1 mg⋅kg^-1^ MK-801 because this dose is sufficient to disrupt PPI (Spooren et al., 2004; Zhang et al., 2007).

#### Experiment 4 - Effect of Buspirone on MK-801-Induced TOR Memory Deficit and Hyperlocomotion in D_3_R^-/-^ Mice

To figure out whether or not the effects of buspirone were mainly mediated via the blockade of D_3_R, we tested D_3_R^-/-^ mice (open field and TOR) treated with the same pharmacological treatment carried out in WT mice, both in terms of doses and timing of treatment. Unfortunately, we could not evaluate the effect of buspirone on PPI test because the vast majority of D_3_R^-/-^ mice exhibited a very low acoustic startle reactivity during the startle matching session (data not shown). This made difficult the assembling of experimental groups with similar PPI%.

### Statistics

Statistical analysis was performed by using graphpad prism 7 (graphpad software La Jolla, CA, United States). In the TOR experiments, one-way ANOVA with *treatment* as between-subject factor was used to determine the main effect. Acoustic startle reactivity was analyzed by performing a two-way ANOVA with *acoustic startle stimulus* as a within-subjects factor and *treatment* as a between-subjects factor. To analyze PPI%, a two-way repeated-measures ANOVA with *prepulse intensity* as a within-subjects factor and *treatment* as a between-subjects factor was carried out. Changes in locomotor activity (number of crossings for each time-point) as well as induction of catalepsy were assessed by performing a two-way repeated-measures ANOVA with *time-point* as a within-subjects factor and *treatment* as a between-subjects factor. A one-way ANOVA with *treatment* as between-subject factor was carried out for the assessment of the total number of crossings. For all data analyses, upon confirmation of significant main effects, differences among individual means were assessed using the Newman–Keuls’ *post hoc* test. For all analyses, significance was accepted with a p value less than 0.05. Standard error of the mean (SEM) and variance were found similar between groups. All data are presented as mean ± SEM.

## Results

### Buspirone Counteracted MK-801-Induced Memory Deficits in WT Mice Tested in the TOR Paradigm

The discrimination performance of WT mice was significantly affected by pharmacological treatments, during the test phase of the TOR test (main effect of *treatment, F*_(5,45)_ = 5.374, *P* = 0.0006, *n* = 8/10 per group). MK-801 induced a strong TOR memory impairment. Indeed, veh + MK-801-treated WT mice exhibited a greater preference in exploring the more recently experienced object in comparison with veh + veh-treated WT mice, which, as expected, spent more time exploring the less recently experienced object (*post hoc* analysis: *P* < 0.001 vs. veh + veh group; **Figure 1B**). Worthy of note, bus + MK-801-treated WT mice explored significantly more the less recently experienced object than the more recently one in a similar manner as veh + veh-treated WT mice (*post hoc* analysis*: P* < 0.01 vs. veh + MK-801 group; *P* > 0.05 vs. veh + veh group; **Figure 1B**). Thus, buspirone efficiently prevented MK-801-induced TOR memory impairment. Clo + MK-801-treated WT mice did not show an optimal discrimination performance even though they performed significantly better than veh + MK-801-treated WT mice and not differently from the veh + veh-treated WT mice (*post hoc* analysis: *P* < 0.05 vs. veh + MK-801 group; *P* > 0.05 vs. veh + veh group; **Figure 1B**). Both buspirone and clozapine, when injected alone, had no effect on discrimination performance (*post hoc* analysis: *P* > 0.05 vs. veh + veh group; **Figure 1C**).

### Buspirone Blocked MK-801-Stimulated Hyperactivity and Did Not Cause Catalepsy in WT Mice

**FIGURE 2:**
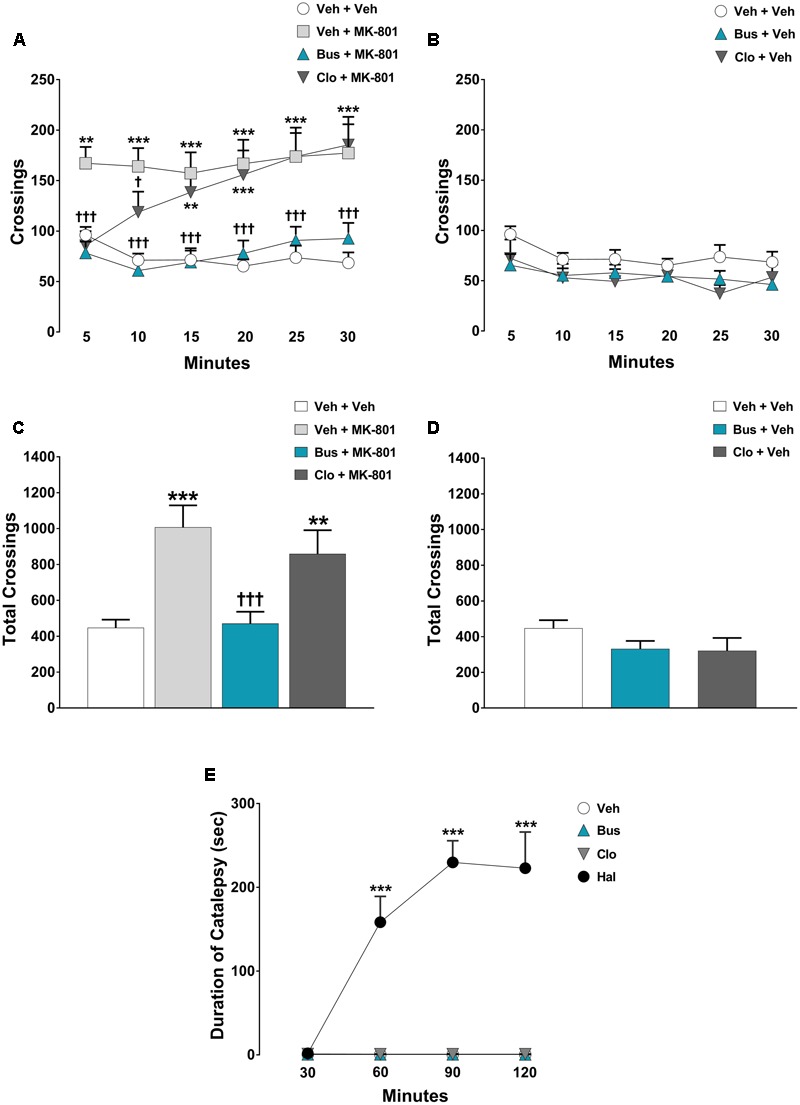
Buspirone blocked MK-801-stimulated hyperlocomotion, but did not cause catalepsy in WT mice. Buspirone (Bus, 3 mg⋅kg^-1^, i.p.) or vehicle (Veh), clozapine (Clo, 1 mg⋅kg^-1^, i.p.) or Veh and MK-801 (0.1 mg⋅kg^-1^, i.p.) or Veh were injected 45 min, 30 min and 20 min respectively, before the open field. **(A,B)** Locomotor activity (crossings) at each 5-min time point displayed by Veh + Veh (*n* = 11), Veh + MK-801 (*n* = 11), Bus + MK-801 (*n* = 10), Clo + MK-801 (*n* = 10), Bus + Veh (*n* = 9), Clo + Veh (*n* = 9) WT mice. **(C,D**) Locomotor activity (crossings) over a 30-min test period displayed by the same mice. **(E)** Duration of catalepsy state 30, 60, 90, and 120 min after drug injection (*n* = 6 animals/group). Haloperidol (Hal, 1 mg⋅kg^-1^) was used as positive control. Data are shown as mean ± SEM. ^∗∗∗^*p* < 0.001, ^∗∗^*p* < 0.01 vs. Veh + Veh WT mice; ^†††^*p* < 0.001 and ^†^*p* < 0.05 vs. Veh + MK-801 WT mice; ^∗∗∗^*p* < 0.001 vs. Veh (Two-way repeated-measures ANOVA and Newman–Keuls *post hoc* test).

The pharmacological treatments significantly modified the locomotor activity of WT mice during each 5-min time-point [main effects of *treatment, F*_(5,54)_ = 10.42, *P* < 0.0001; *time-point, F*_(5,270)_ = 4.274, *P* = 0.0009; *treatment* × *time-point* interaction, *F*_(25,270)_ = 6.18, *P* < 0.0001; *n* = 9/11 per group]. In addition, ANOVA showed a significant main effect of *treatment* [*F*_(5,54)_ = 10.42, *P* < 0.0001] on the total crossings over 30 min for WT mice. As expected, MK-801 produced a strong hyperlocomotion in WT mice. Indeed, veh + MK-801-treated WT mice performed a significant higher number of crossings compared to veh + veh-treated WT mice (*post hoc* analysis: 5-min: *p* < 0.01; from 10-min to 30-min *p* < 0.001 **Figures 2A,C**). Interestingly, buspirone did not alter *per se* the locomotor activity (*post hoc* analysis: all time-points *p* > 0.05 vs. veh + veh group; **Figures 2B,D**), but it completely blocked MK-801-induced hyperactivity. Bus + MK-801-treated WT mice performed a significant lower number of crossings compared to veh + MK-801-treated WT mice (*post hoc* analysis: all time-points: *p* < 0.001 vs. veh + MK-801 group; **Figures 2A,C**), displaying a locomotor activity similar to that of veh + veh-treated WT mice (*post hoc* analysis: All time-points: *p* > 0.05 vs. veh + veh group; **Figures 2A,C**). Clozapine did not modify *per se* the locomotor activity (*post hoc* analysis: all time-points: *p* > 0.05 vs. veh + veh group; **Figures 2B,D**), but it significantly prevented MK-801-induced hyperactivity only in the first 10 min, loosing progressively its efficacy from the 15-min time point to the end of the test (*post hoc* analysis: 5-min: *p* < 0.001; 10-min: *p* < 0.05 vs. veh + MK-801 group. 15-min: *p* < 0.01; from 20-min to 30-min *p* < 0.001 vs. veh + veh group; **Figures 2A,C**).

Regarding the catalepsy test, significant main effects of *treatment* [*F*_(3,20)_ = 48.11, *P* < 0.0001, *n* = 6 per group] and *time-point* [*F*_(3,60)_ = 21.70, *P* < 0.0001], together with a significant *treatment* × *time-point* interaction [*F*_(3,60)_ = 21.77, *P* < 0.0001] were found on the duration of catalepsy. As expected, haloperidol caused a severe catalepsy state (*post-hoc* analysis: all time-points: *P* < 0.001 vs. veh group; **Figure 2E**), an effect not induced by clozapine or buspirone (*post hoc* analysis: all time-points: *P* > 0.05 vs. veh group; **Figure 2E**).

### Buspirone Blocked MK-801-Induced PPI Disruption in WT Mice

**FIGURE 3:**
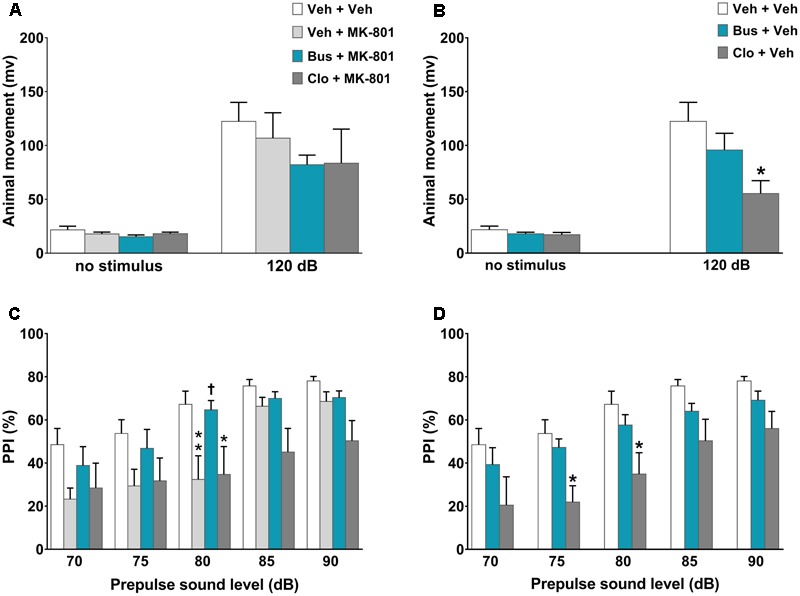
Buspirone blocked MK-801-induced PPI disruption in WT mice. Buspirone (Bus, 3 mg⋅kg^-1^, i.p.) or vehicle (Veh), clozapine (Clo, 1 mg⋅kg^-1^, i.p.) or Veh and MK-801 (0.1 mg⋅kg^-1^, i.p.) or Veh were injected 45, 30, and 20 min before the PPI test, respectively. **(A,B)** Animal movements displayed by Veh + Veh (*n* = 10), Veh + MK-801 (*n* = 10), Bus + MK-801 (*n* = 9), Clo + MK-801 (*n* = 8), Bus + Veh (*n* = 13), Clo + Veh (*n* = 8) WT mice. **(C,D)** PPI% displayed by the same WT mice. Data are shown as mean ± SEM. ^∗∗^*p* < 0.01,^∗^*p* < 0.05 vs. Veh + Veh WT mice; ^†^*p* < 0.05 vs. Veh + MK-801 WT mice; (Two-way ANOVA with or without repeated-measures and Newman–Keuls *post hoc* test).

In the assessment of acoustic startle reactivity, ANOVA revealed a main effect of *acoustic startle stimulus* [*F*_(1,110)_ = 76.42, *P* < 0.0001, *n* = 8/11 per group] but not a main effect of *treatment* [*F*_(5,110)_ = 1.36, *P* = 0.2450] or a significant interaction between the factors [*F*_(5,110)_ = 1.048, *P* = 0.3933]. Except for clozapine, which *per se* significantly decreased the acoustic startle reactivity at 120-dB (*post hoc* analysis at120 dB stimulus: *P* < 0.05 vs. veh + veh group; **Figure 3B**), all other experimental groups displayed similar acoustic startle reactivity (*post hoc* analysis: *P* > 0.05 vs. veh + veh group; **Figures 3A,B**). With regard to the PPI test, there were significant main effects of *treatment* [*F*_(5,55)_ = 3.525, *P* = 0.0078] and *prepulse intensity* [*F*_(4,220)_ = 49.16, *P* < 0.0001] but not a significant *treatment* × *prepulse intensity* interaction [*F*_(20,220)_ = 1.4, *P* = 0.1238]. As expected, MK-801 significantly disrupted PPI; veh + MK-801-treated WT mice showed a progressively lower PPI% that reached statistical significance at 80 dB prepulse intensity, (*post hoc* analysis: *P* < 0.01 vs. veh + veh group; **Figure 3C**). Interestingly, buspirone, which had no effect on PPI when administered alone (*post hoc* analysis: *P* > 0.05 vs. veh + veh group; **Figure 3D**), completely blocked MK-801-induced PPI disruption. Bus + MK-801-treated WT mice exhibited PPI%, significantly greater than veh + MK-801-treated WT mice at 80 dB prepulse intensity, and similar to veh + veh-treated WT mice at all prepulse intensities (*post hoc* analysis*: P* < 0.01 vs. veh + MK-801 group, *p* > 0.05 vs. veh + veh group; **Figure 3C**). Noteworthy, clozapine *per se* disrupted PPI (*post hoc* analysis: 75 and 80 dB prepulse: *p* < 0.05 vs. veh + veh group; **Figure 3D**), but did not block MK-801-induced PPI disruption (*post hoc* analysis at 80 dB prepulse: *p* < 0.05 vs. veh + veh group and *p* > 0.05 vs. veh + MK-801 group; **Figure 3C**).

### Buspirone Was Ineffective in Preventing MK-801-Induced TOR Memory Deficit and Scarcely Effective in Counteracting MK-801-Stimulated Hyperlocomotion in D_3_R^-/-^ Mice

**FIGURE 4:**
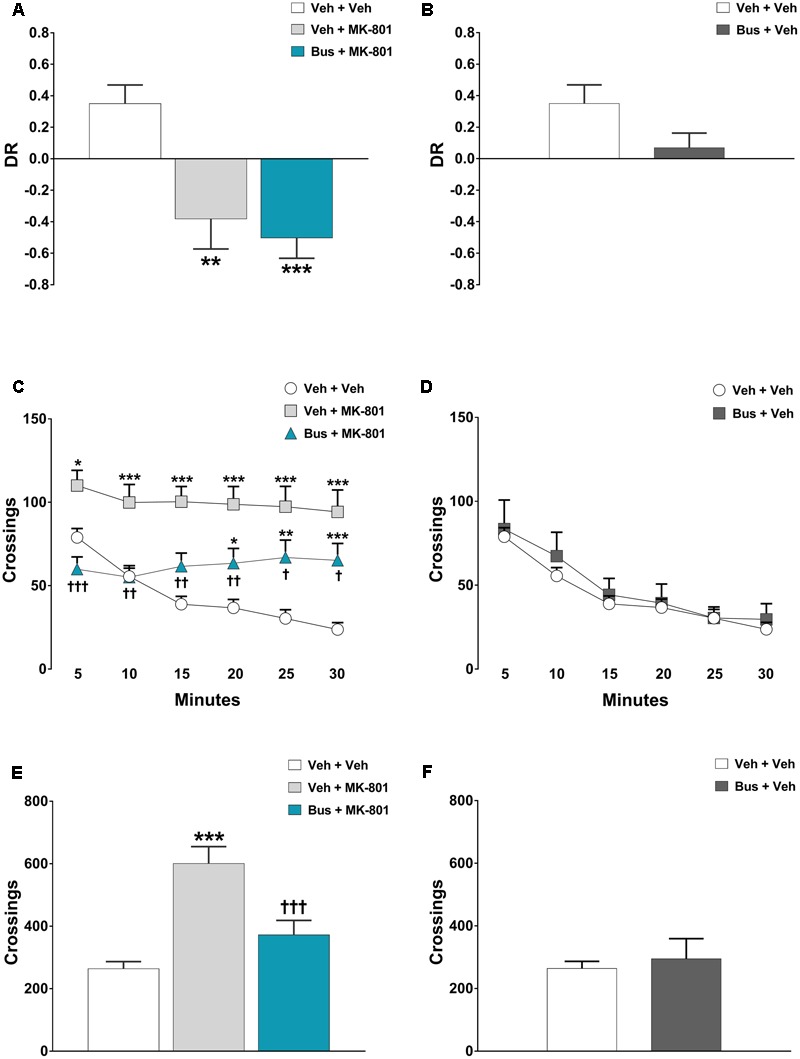
Buspirone was ineffective in preventing MK-801-induced TOR memory deficit and hyperlocomotion. Buspirone (Bus, 3 mg⋅kg^-1^, i.p.) or vehicle (Veh) and MK-801 (0.1 mg⋅kg^-1^, i.p.) or Veh were injected 45 min, and 20 min respectively, before the sample phase 2 or the open field test. **(A,B)** Discrimination ratio (DR) displayed by Veh + Veh (*n* = 5), Veh + MK-801 (*n* = 6), Bus + MK-801 (*n* = 5), Bus + Veh (*n* = 6), D_3_R^-/-^ mice during the test phase. DR [(less recently experienced object exploration time - more recently experienced object exploration time)/total exploration time]. **(C,D)** Locomotor activity (crossings) at each 5-min time point displayed by Veh + Veh (*n* = 11), Veh + MK-801 (*n* = 11), Bus + MK-801 (*n* = 11), Bus + Veh (*n* = 6), D_3_R^-/-^ mice. **(E,F)** Locomotor activity (crossings) over a 30-min test period displayed by the same mice. Data are shown as mean ± SEM. ^∗∗∗^*p* < 0.001, ^∗∗^*p* < 0.01, ^∗^*p* < 0.05 vs. Veh + Veh D_3_R^-/-^ mice; ^†††^*p* < 0.001, ^††^*p* < 0.01 and ^†^*p* < 0.05 vs. Veh + MK-801 WT mice; (One-way or two-way repeated-measures ANOVA and Newman–Keuls *post hoc* test).

The memory of D_3_R^-/-^ mice was significantly affected by pharmacological treatments, during the test phase of the TOR test [main effect of *treatment F*_(3,18)_ = 7.478, *P* = 0.0019, *n* = 5/6 per group]. MK-801 produced a marked TOR memory deficit in D_3_R^-/-^ mice, comparable to that observed in WT mice. In particularly, veh + MK-801-treated D_3_R^-/-^ mice significantly preferred exploring the more recently experienced object than the less recently one in contrast with veh + veh-treated D_3_R^-/-^ mice that displayed an intact TOR memory and behaved in the opposite way (*post hoc* analysis: *P* < 0.01 vs. veh + veh D_3_R^-/-^ group; **Figure 4A**). Consistent with the hypothesis that buspirone acts on D_3_R receptors, bus + MK-801-treated D_3_R^-/-^ mice behaved in a manner similar to veh + MK-801-treated D_3_R^-/-^, showing the same TOR memory impairment (*post hoc* analysis: *P* < 0.001 vs. veh + veh D_3_R^-/-^ group, *P* > 0.05 vs. veh + MK-801 D_3_R^-/-^; **Figure 4A**), i.e., in D_3_R^-/-^ buspirone was unable to prevent MK-801-induced TOR memory impairment as it did in WT mice. Notice that at variance with what observed in WT mice, in D_3_R^-/-^ buspirone on its own disrupted the discrimination of the experienced objects, though not in a significant manner (*post hoc* analysis: *P* > 0.05 vs. veh + veh D_3_R^-/-^ group; **Figure 4B**).

In the OF test, MK-801 produced also a robust and persistent hyperlocomotion in D_3_R^-/-^ mice; significant main effects of *treatment* [*F*_(3,35)_ = 11.74, *P* < 0.0001, *n* = 8/11 per group] and *time-point* [*F*_(5,175)_ = 15.92, *P* < 0.0001], and a significant *treatment* x *time-point* interaction [*F*_(15,175)_ = 5.123, *P* < 0.0001] were found on locomotor activity of D_3_R^-/-^ mice during each 5-min time-point. Moreover, ANOVA revealed a significant main effect of *treatment* [*F*_(3,35)_ = 11.34, *P* < 0.0001] on the total crossings that D_3_R^-/-^ mice performed throughout the 30 min of the test. Veh + MK-801-treated D_3_R^-/-^ mice, compared to veh + veh-treated D_3_R^-/-^ mice, carried out a significant higher number of crossings (*post hoc* analysis at 5-min: *p* < 0.05; from 10-min to 30-min: *p* < 0.001; **Figures 4C,E**). Buspirone, which was devoid of effect when injected alone (*post-hoc* analysis: all time-points *p* > 0.05 vs. veh + veh D_3_R^-/-^ group; **Figures 4D,F**), significantly attenuated MK-801-stimulated hyperlocomotion, but its effect diminished from the 10-min time point on. Indeed, Bus + MK-801-treated D_3_R^-/-^ mice performed a number of crossings similar to that of veh + veh-treated D_3_R^-/-^ mice but significantly lower compared to veh + MK-801-treated D_3_R^-/-^ mice during the first 10 min (*post hoc* analysis at 5-min: *p* < 0.001; from 10-min to 20-min: *p* < 0.01; at 25-min and 30-min: *p* < 0.05 vs. veh + MK-801 D_3_R^-/-^ group; **Figures 4C,E**).

## Discussion

These results provide the first evidence that buspirone counteracts a wide-range of schizophrenia-relevant phenotypes through its antagonism at D_3_R.

To investigate the antipsychotic properties of buspirone, we chose a pharmacological model based on NMDAR hypofunction triggered by acute administration of the NMDAR antagonist MK-801. Although not devoid of limitations, this model is extensively employed for the assessment of potential antipsychotic activity of investigational compounds (Bubeníková-Valesová et al., 2008; Adell et al., 2012). Indeed, NMDAR dysfunction may recapitulate “core” symptoms of schizophrenia, particularly the multiplicity of cognitive deficits, more faithfully than dopamine-based models (Kantrowitz and Javitt, 2010).

Cognitive deficits observed in schizophrenic patients have been strongly associated with an abnormal PFC activity (Driesen et al., 2008). Earlier studies indicated that the cognitive impairment induced by MK-801 arises from an intensification of the discharge of mPFC pyramidal neurons, triggered via NMDAR blockade in inhibitory interneurons of mPFC and hippocampus (HP, Homayoun et al., 2005; Jodo et al., 2005; Homayoun and Moghaddam, 2007). D_3_Rs are expressed specifically in layer 5 pyramidal neurons of mPFC of both primate and rodents (Lidow et al., 1998) and uniquely modulate the neuronal excitability (Clarkson et al., 2017). Consequently, D_3_Rs play a fundamental role in prefrontal-dependent cognitive functions (Nakajima et al., 2013). Studies on dopamine receptor-specific reporter gene mice further revealed an abundant expression of D_3_Rs in HP^1^; furthermore, hippocampal lesions leave single item object recognition memory intact, while impair temporal order memory (Warburton and Brown, 2015). Based on these premises, we assessed the effect of buspirone in the TOR memory task. This behavioral task depends on interconnections among mPFC, perirhinal cortex (PRH) and HP (Barker and Warburton, 2011; Managò et al., 2016) and is used to measure recency discrimination, a cognitive function impaired in schizophrenic patients (Schwartz et al., 1991; Rizzo et al., 1996). To our knowledge, this is the first study demonstrating that acute systemic administration of MK-801 at the dose of 0.1 mg⋅kg^-1^, markedly impairs TOR memory in mice. Therefore, our results confirm the face validity of the pharmacological model based on NMDAR hypofunction triggered by acute administration of the NMDAR antagonist MK-801, being also consistent with earlier findings showing a disruption of TOR memory following intra-PRH or intra-mPFC infusion of the selective NMDAR antagonist AP5 (Warburton et al., 2013). We found that buspirone prevented MK-801-induced TOR memory impairment in WT mice even better than clozapine. Very interestingly, this effect was completely abolished in D_3_R^-/-^ mice. Thus, these data provide the first evidence that buspirone may be effective in treating cognitive deficits in schizophrenia, and that its efficacy against MK-801-induced cognitive dysfunction relies exclusively on D_3_Rs blockade. These findings are particularly relevant, considering that cognitive dysfunction represents a major challenge in the pharmacological treatment of schizophrenic patients. Furthermore, our results are consistent with previous studies, reporting that some antipsychotics that behave as selective D_3_R antagonists or D_3_R preferring partial agonists enhance cognitive functions in schizophrenia (Nakajima et al., 2013; Zimnisky et al., 2013). None of the available antipsychotics is truly selective for D_3_R (Schotte et al., 1996; McCormick et al., 2010); as a result, current drug treatments generally improve positive symptoms (delusions, hallucinations), but poorly change negative symptoms (lack of motivation, social withdrawal, anhedonia) or cognitive dysfunction. The present data obtained with buspirone reinforce the view that blockade of D_3_R may improve cognition, which represents a translational potential for schizophrenia treatment.

Recently, Barker et al. (2017) discovered that the pharmacogenetic deactivation of a specific neuronal circuit originating in the dorsal CA1 region of HP and projecting to mPFC, selectively disrupts TOR memory in mice. Thus, we speculate that a glutamatergic/dopaminergic imbalance in specific neuronal circuits connecting HP and mPFC might disrupt the connection between these two brain areas, leading to memory impairment in mice tested in the TOR paradigm. In this context, D_3_R blockade, particularly in mPFC and HP, might prevent the hyperactivity of the dopaminergic system subsequent to NMDARs hypofunction (Snyder and Gao, 2013). However, because D_3_R^-/-^ mice appeared to be as sensitive as WT mice to the cognitive effects of acute administration of MK-801, other neurotransmitters and/or dopamine receptor subtypes are likely to be involved, and may represent compensatory mechanisms that prevails over D_3_R control in D_3_R^-/-^ mice.

Hyperactivity is a valuable correlate, easily modeled in rodents, widely associated with positive symptoms and psychomotor agitation in most schizophrenic patients (Jones et al., 2011). Here, we found that buspirone blocked MK-801-stimulated hyperactivity, but did not cause catalepsy in WT mice; moreover, because the preventing effect of buspirone on MK-801-stimulated hyperactivity was not very strong in D_3_R^-/-^ mice, it must be, at least in part, attributable to D_3_R antagonism. This conclusion is consistent with earlier studies showing D_3_R antagonists as effective on MK-801-stimulated hyperactivity (Leriche et al., 2003; Brindisi et al., 2014; Sun et al., 2016). Considering that positive symptoms are not well-managed in a considerable number of patients suffering from schizophrenia (Miyamoto et al., 2012), our observation, together with other published reports, points to D_3_R as potential target to treat hyperactivity.

We cannot exclude a contribution of other receptors targeted by buspirone in mediating its antipsychotic-like effects in our experimental paradigms. Buspirone in fact, binds to 5-HT_1A_R, where it behaves as potent partial agonist (Bergman et al., 2013), and several studies have reported that 5-HT_1A_R antagonists or partial agonists attenuate psychotomimetic effects of MK-801 (Wedzony et al., 2000; Park et al., 2005; Bubenikova-Valesova et al., 2010). Furthermore, buspirone also binds to D_4_R with high affinity and behaves as antagonist (Bergman et al., 2013). A highly selective dopamine D_4_R antagonist was found to decrease amphetamine-induced hyperlocomotion (Boeckler et al., 2004). Consequently, we cannot exclude a contribute of D_4_R in the effects we reported here.

PPI is a valuable model to study the sensorimotor gating disruption classically observed in schizophrenia (Papaleo et al., 2012). Because animals and humans are tested in a similar way, this model has face, construct, and predictive validity and is widely employed to identify potential antipsychotic properties of recently developed drugs (Rigdon and Viik, 1991). Our findings demonstrated that buspirone, devoid of effect by itself, completely counteracted PPI disruption dependent on NMDAR hypofunction. These results are partially in agreement with previous studies showing that buspirone weakly counteracts apomorphine-induced PPI disruption (Rigdon and Viik, 1991) while it was without effect on its own (Van den Buuse and Gogos, 2007). The antipsychotic-like effect of buspirone on MK-801-induced PPI disruption might be mainly driven by its antagonist activity at D_3_R. Several reports proved that selective D_3_R antagonists improve PPI disruption in different preclinical models of schizophrenia (Zhang et al., 2006; Maramai et al., 2016; Sun et al., 2016). Unfortunately we could not directly address the D_3_R involvement on the buspirone’s effect in PPI by using D_3_R^-/-^, because these mice did not exhibit a robust acoustic startle reactivity, suitable for making reliable measurements. However, it is unlikely that the 5 HT_1A_R partial agonist activity of buspirone could contribute to its efficacy on MK-801-induced PPI disruption. Bubenikova-Valesova et al. (2010) found the selective 5-HT_1A_R partial agonist tandospirone exacerbates MK-801-induced PPI disruption and other groups reported a PPI disruption after 5-HT_1A_R stimulation (Rigdon and Weatherspoon, 1992; Gogos and Van den Buuse, 2003; Gogos et al., 2006). Again, we cannot exclude the possible involvement of the D_4_R blockade also in the effect of buspirone in MK-801-induced PPI disruption. However, contrasting results have shown positive/negative effects of D_4_R antagonists in ameliorating apomorphine-induced PPI disruption (Bristow et al., 1997; Mansbach et al., 1998; Boeckler et al., 2004). Worthy of note, clozapine did not prevent MK-801-induced PPI disruption or MK-801-stimulated hyperlocomotion. Considering that clozapine is one of the most effective antipsychotic drugs, the discrepancy with its poor efficacy in preclinical models point once more to the need for defining “gold pharmacological standards” preclinical models of schizophrenia (Jones et al., 2011), taking into account that doses, strains, behavioral paradigms, all affect the variability, reproducibility and translationality to clinical settings.

## Conclusion

The present study demonstrates that buspirone, a drug currently approved for the treatment of anxious disorders, might be a potential antipsychotic medication and also that D_3_R represents a valuable pharmacological target especially for the treatment of cognitive deficits in schizophrenia. Anxious symptoms and cognitive impairment frequently co-occur especially in the prodromal phase of the disease, when the positive symptoms are below the threshold for psychosis (Corigliano et al., 2014). In this scenario, buspirone might represent a new pharmacological tool to treat the early phase of the disease. Indeed, the early intervention is the best way to prevent development of chronic disabilities. Finally, these findings are particularly relevant considering that a substantial number of pharmaceutical industries are turning away from developing antipsychotics for many reasons, including costs, unclear disease mechanisms and long-lasting developmental processes. Repositioning of buspirone and/or of other drugs endowed with D_3_R antagonist activity, could therefore open new avenues to foster schizophrenia drug treatments. However, further studies are needed to evaluate the efficacy of this drug after chronic treatment in an animal model provided with the three criteria of face, construct and predictive validity.

## Author Contributions

ST, SS, and GL designed research; ST, FG, and GL performed research; FC and CB helped with some experiments; ST and GL analyzed data; FD, SS, and GL supervised the work; ST and GL wrote the manuscript.

## Conflict of Interest Statement

The authors declare that the research was conducted in the absence of any commercial or financial relationships that could be construed as a potential conflict of interest.

## Footnotes

1 www.gensat.org
